# OlfactionBase: a repository to explore odors, odorants, olfactory receptors and odorant–receptor interactions

**DOI:** 10.1093/nar/gkab763

**Published:** 2021-09-01

**Authors:** Anju Sharma, Bishal Kumar Saha, Rajnish Kumar, Pritish Kumar Varadwaj

**Affiliations:** Department of Applied Science, Indian Institute of Information Technology, Allahabad, Uttar Pradesh 211015, India; Gentryx Technologies, West Bengal, Kolkata, India; Amity Institute of Biotechnology, Amity University Uttar Pradesh, Lucknow Campus, Uttar Pradesh 226028, India; Department of Applied Science, Indian Institute of Information Technology, Allahabad, Uttar Pradesh 211015, India

## Abstract

Olfaction is a multi-stage process that initiates with the odorants entering the nose and terminates with the brain recognizing the odor associated with the odorant. In a very intricate way, the process incorporates various components functioning together and in synchronization. OlfactionBase is a free, open-access web server that aims to bring together knowledge about many aspects of the olfaction mechanism in one place. OlfactionBase contains detailed information of components like odors, odorants, and odorless compounds with physicochemical and ADMET properties, olfactory receptors (ORs), odorant- and pheromone binding proteins, OR-odorant interactions in Human and *Mus musculus*. The dynamic, user-friendly interface of the resource facilitates exploration of different entities: finding chemical compounds having desired odor, finding odorants associated with OR, associating chemical features with odor and OR, finding sequence information of ORs and related proteins. Finally, the data in OlfactionBase on odors, odorants, olfactory receptors, human and mouse OR-odorant pairs, and other associated proteins could aid in the inference and improved understanding of odor perception, which might provide new insights into the mechanism underlying olfaction. The OlfactionBase is available at https://bioserver.iiita.ac.in/olfactionbase/.

## Graphical Abstract

**Graphical Abstract:**
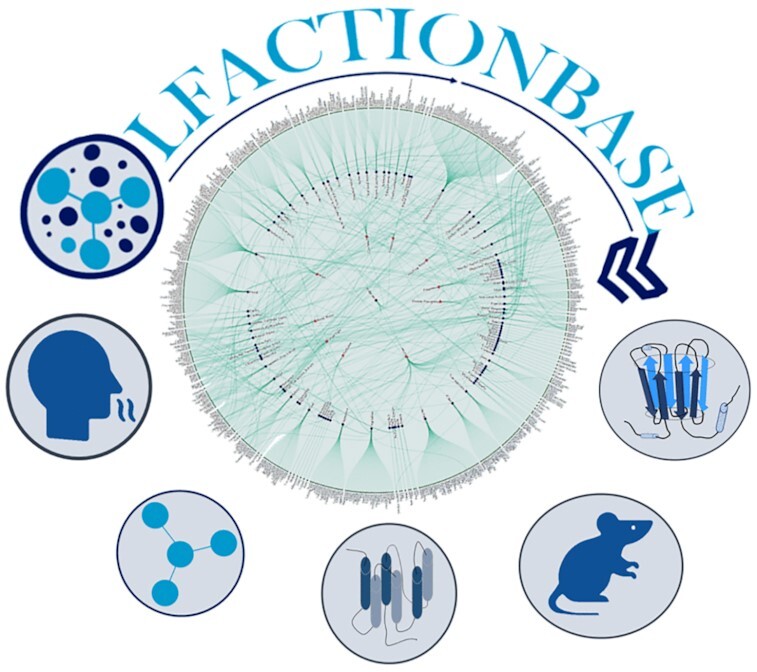
OlfactionBase contains information about odors, odorant, olfactory receptors and odorant-receptor interaction.

## INTRODUCTION

Olfaction is a complex, most primeval, volatile, and in comparison, to other senses, less explored multi-sensory mechanism which influences our innate response (1–7). It is highly dynamic, deceptive and complex, capable of detecting and distinguishing between a wide range of small volatile, lighter, hydrophobic compounds (odorants) with diverse chemical and structural properties, even at a very low concentration (parts per trillion) (8–13). The perception of olfaction arises from the interaction of odorants with the highly specific biological machinery, i.e., ORs present in the nasal/ olfactory epithelium (OE) (14–20). A unique pattern of neuronal signals is produced for each odorant, consisting of signal intensity, time, and quality of odorant stimuli which further stimulates a specific population of olfactory sensory neurons (OSNs) present in OE, which process and transduce the signals at the neurological level (21–34). The perception of neural signals produces a representation known as ‘smell/ odor,’ which is semantically represented by various perceptual descriptors such as fruity, rose, woody, etc.

The challenge associated with the olfaction process is daunting because odors are insubstantial, have a complex molecular basis, and are perceived individually (35–39). Odors associated with any substance (flower, plant, etc.) are made up of various odorants, some of which are significant contributors, and others are minor contributors. For example, the main constituent of rose smell is (−)-*cis* rose oxide, while the minor constituent is beta-damascenone, farnesol, geraniol, etc. The relationship between odorant chemistry and odor perception is the core of olfaction research. Since an odorant can have several odors (eugenol methyl ether associated with 27 odor perceptions), two structurally different chemicals can have virtually the same odor profile (*cis*-3-hexenol, nonadienal, ligustral exhibits green odor). A slight structural difference can result in distinct odors (carvone enantiomers, (*R*)-(−)-carvone (spearmint odor) and (*S*)-(+)-carvone (caraway odor)); this complex relationship is largely unknown until today. Odors are further encoded using a combinatorial approach, in which structurally identical odorants bind to completely different but overlapping olfactory receptors (ORs), hence significantly increasing the problem's complexity (40,41). There is no adequate scientific explanation for how smell is interpreted, particularly in humans. Although some pieces of the puzzle have been discovered in certain species, we still lack a comprehensive understanding of the phenomenon (42,43).

Several databases for odors, chemical compounds, and ORs have been published, but they are still limited in scope and concentrate on specific aspects (Table 1). Some resources focus solely on odorant compounds, others on odorant−OR pairs, and others on odors alone. As a result, these databases are fine but only useful for specific purposes, necessitating creating a robust website with all knowledge available at a single click. Also, there is no database available for odorless compounds. In this study, we created OlfactionBase with the aim of integrating multidimensional aspects of significant components involved in the olfaction mechanism, i.e., odors, chemicals (odorants and odorless), ORs, odorant−ORs interaction, and other associated proteins (odorant-binding proteins (OBPs), pheromone binding proteins (PBPs), and chemosensory proteins). OlfactionBase is a manually curated comprehensive database collating information from various resources, comprised of 106 primary odors, 572 subodor types, 3985 odorant molecules, 1124 odorless compounds, 2067 ORs (human and *Mus musculus*) compiled from various sources. OlfactionBase stores 874 (408 Human and 466 mouse) odorant−OR interaction information, thereby provides a platform for comparative analysis and quantitative structure odor relationship (QSOR) studies. All the data was manually compiled and extracted from the literature and database searches.

**Table 1.** Summary of different databases related to olfaction.

Content type	Database	Description	Website address
Odors and Odorants	AroChemBase	A database of compounds for aroma and chemical analysis using gas chromatography and Kovats index calculations.	http://www.alpha-mos.com/
	AromaDB (44)	A database of medicinal and aromatic plant's aroma.	http://bioinfo.cimap.res.in/aromadb/
	EssOilDB (45)	A database of plants essential oils reflecting terpene composition and GC/MS data.	http://nipgr.res.in/Essoildb/
	FlavorBase	A database of flavoring materials and food additives.	http://www.leffingwell.com/flavbase.htm
	Flavordb (46)	A database of flavor molecules.	https://cosylab.iiitd.edu.in/flavordb/
	Flavornet (47)	A database of volatile compounds related to human olfaction.	http://www.flavornet.org/flavornet.html
	mVOC (48)	A database of microbial volatiles.	http://bioinformatics.charite.de/mvoc
	Pubchem (49)	An open chemistry database for small and large molecules with information on chemical structures, chemical, and physical properties, and many others.	https://pubchem.ncbi.nlm.nih.gov/
	ScentBase (50)	A database of floral scent components.	http://www2.dpes.gu.se/SCENTbase.html/
	Sigma Aldrich (51)	A database for flavors, fragrances, and associated chemicals.	https://www.sigmaaldrich.com/industries/flavors-and-fragrances.html
	SuperScent (52)	A database of volatille compounds associated with scents, including synthetic compounds.	http://bioinf-applied.charite.de/superscent/
	TGSC	A database of flavor, fragrance, food, and cosmetic industries.	http://www.thegoodscentscompany.com/
Olfactory receptors	HORDE (53)	A database of human olfactory receptors.	https://genome.weizmann.ac.il/horde/
	ORDB (54)	A database of vertebrate OR genes and proteins.	https://senselab.med.yale.edu/ordb/
	UniProt (55)	A comprehensive resource of protein sequence and functional information.	https://www.uniprot.org/
Odorant-OR interaction	OdoRactor (56)	A web server on odorant identification and olfactory repertoire browse.	http://mdl.shsmu.edu.cn/ODORactor/
	OlfactionDB (57)	A database of olfactory receptors and their Ligands	http://molsim.sci.univr.it/OlfactionDB.

**Figure 1.**
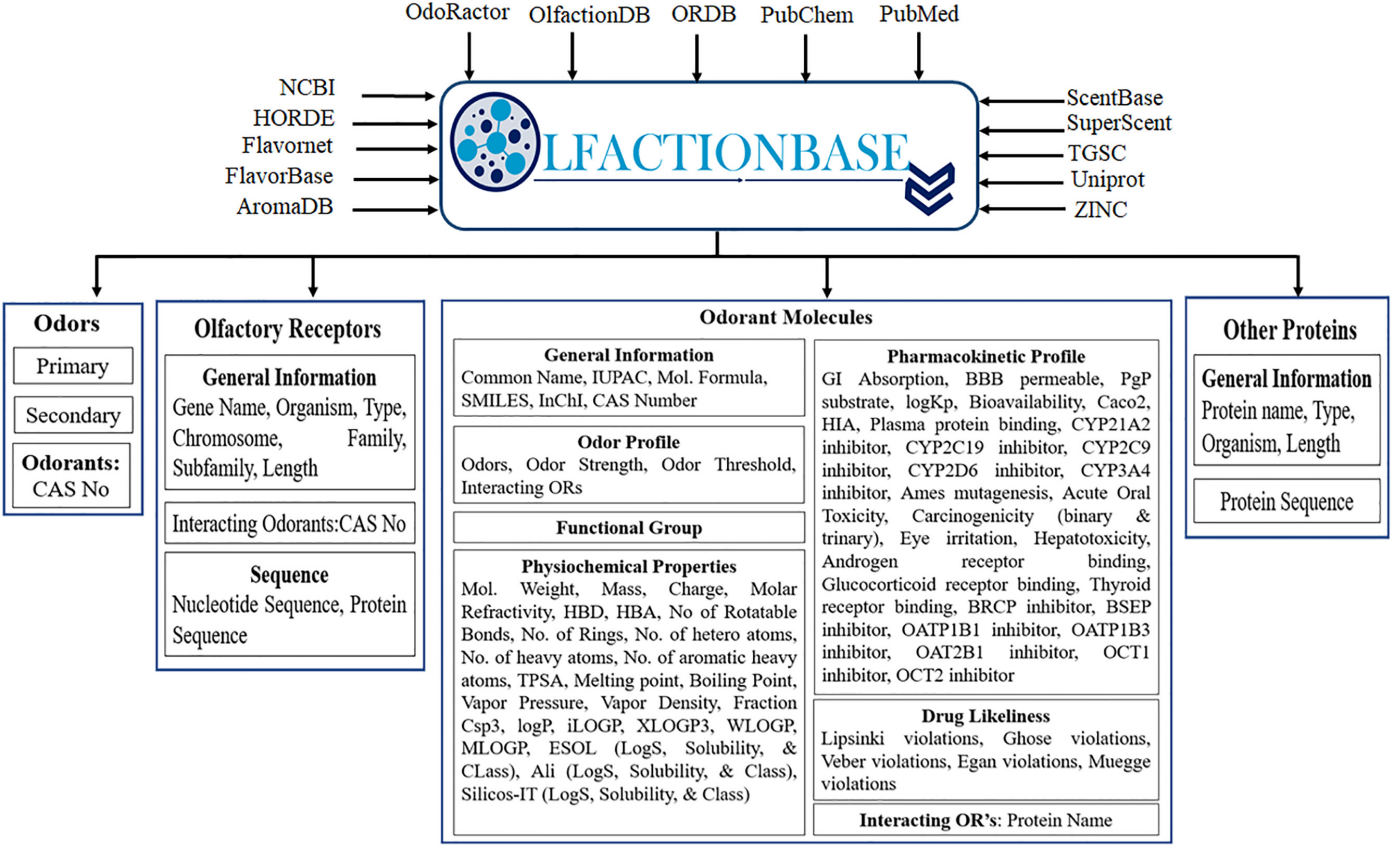
OlfactionBase database architecture. OlfactionBase offers a robust dataset of odors, odorants, olfactory receptors information, user-friendly interface and interlinked search options.

One feature that sets OlfactionBase apart from similar resources is that it presents information in a hierarchy of primary odors, sub-odors, odorants with their odor profile, interacting ORs, and chemical profile, including physicochemical, functional groups, ADMET (absorption, distribution, metabolism, excretion, toxicity) properties. The hierarchy is represented dynamically in the form of Olfaction-Wheel, which offers an integrated platform to explore odor-space, starting from the aroma-wheels suggested by different researchers and terminating at chemicals associated with an odor along with their interacting ORs and chemical information. Hence, OlfactionBase combines different dimensions of odorants constituting the ‘odor space’, ‘interaction space’ and ‘properties space’. Similarly, the general profile of ORs includes sequence, family, organism, length, and interacting odorants. OlfactionBase also houses 2871 entries related to odorant/pheromone binding and chemosensory proteins. OlfactionBase offers a robust dataset backed by an innovative visualization, user-friendly interface, and inter-linked search options for exploring components related to olfaction research at a single platform (Figure 1). OlfactionBase thus paves the way for a better understanding of odor perception due to the dynamic interplay between odors, odorant molecules and their properties with olfactory receptors interaction information, leading to future research directions for scientists.

## DATABASE OVERVIEW

OlfactionBase is a repository with extensive coverage of 5109 chemicals, 2067 ORs, 874 OR-odorant pairs, 106 primary odors and 572 subodors. The chemicals data comprises 3985 odorant and 1124 odorless compounds. Further, the chemical molecules are classified into 30 functional groups and mapped to 572 subodors. OlfactionBase houses the information of ORs from two organisms, i.e., human (851) and *Mus musculus* (1215). It also lists 2871 OBP/ PBP protein information from 190 species.

OlfactionBase has a simple, user-friendly, and intuitive interface for querying and browsing odors, chemicals, and ORs. Interactive data visualizations such as the Olfaction wheel and interlinked textual and drawing-based (for chemical structure) search options are provided to retrieve relevant information. The Olfaction Wheel allows the user to interactively browse, backward and forward, through the odor classifications to access corresponding odorant molecules and subsequently obtain details of the odorant's chemical profile and interacting ORs. Thus, OlfactionBase provides a broad spectrum of information facilitating insights into the olfaction research through dynamic interface and visualizations.

### DATA COMPILATION

The aim of developing OlfactionBase was to organize all information related to olfaction machinery under one umbrella. A list of odors and sub-odors was created from nine classification systems. The overlapping information between classification systems was manually examined and classified into 106 primary odors and 572 subodors.

Each chemical molecule was mapped to 30 functional groups. We provided the standard CAS (Chemical Abstract Service) for each chemical mapped to their corresponding Pubchem and ZINC IDs. Since CAS numbers are degenerate and often point to multiple molecules, PubChem IDs were used as the unique primary key for every chemical. Using PubChem Id, compounds identifiers (such as IUPAC name, common name, physicochemical properties, 2D images, and structure (.mol) files) were obtained from PubChem FTP service. The odor profile of odorants was created by compiling information and mapped using data collected from AromaDB (44), FlavorBase (http://www.leffingwell.com/flavbase.htm), Flavornet (47), PubChem (49), Sigma Aldrich (51), SuperScent (52), TGSC (http://www.thegoodscentscompany.com/), Odoractor (56), and OlfactionDB 57), followed by redundancy removal. Further ADMET, few physicochemical properties and Drug likeness for all 5109 chemicals were obtained from admetsar2 web server (58).

**Figure 2.**
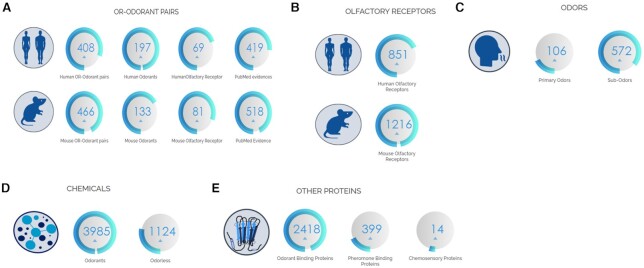
Statistics of OlfactionBase. (**A**) Numbers of OR-odorant pairs in humans and mouse, (**B**) numbers of olfactory receptors, (**C**) numbers of odors, (**D**) numbers of chemicals, (**E**) number of other proteins related to olfaction.

The information of human and mouse ORs was collected from UniProt, HORDE, ORDB database. We provide its UniProt ID, GenBank Accession number, ORDB, and HORDE identifier, and links to respective databases for each receptor. Using UniProt IDs, receptor information such as length, family, subfamily, chromosome number, protein, and nucleotide sequences were obtained. The other proteins related to olfaction are collected using keyword search (‘odorant-binding protein’, chemosensory protein, and pheromone binding protein) in the UniProt database. The OR-odorant interaction information was collected from an extensive literature survey (59–90) and database search. The supporting literature evidence from PubMed for odorant-OR pairs was provided for each entry. Figure 2 refers to the statistics of entities of OlfactionBase.

## DATABASE ARCHITECTURE AND WEB INTERFACE

**Figure 3.**
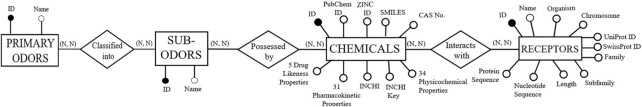
Relational scheme. Each type of entry, i.e., odor, chemical, or receptor, is represented by a different field, depending on the nature of the molecule. Odors are linked to chemicals, while chemicals are linked to receptors using the interaction information from published literature.

OlfactionBase is a free, open-access web server powered by MySQL relational database management system. For better performance, data is kept in interrelated tables logically (Figure 3). All the tables are optimized to serve their purpose. The webserver has been built using the PHP Laravel framework, to deploy dynamic pages onto the server. Two JavaScript libraries (D3.js and Vue.js) have been used to develop the Olfaction Wheel and inner graphs. Other applications like composer, git, CSS3 and HTML were also used to create the entire web application of OlfactionBase.

**Figure 4.**
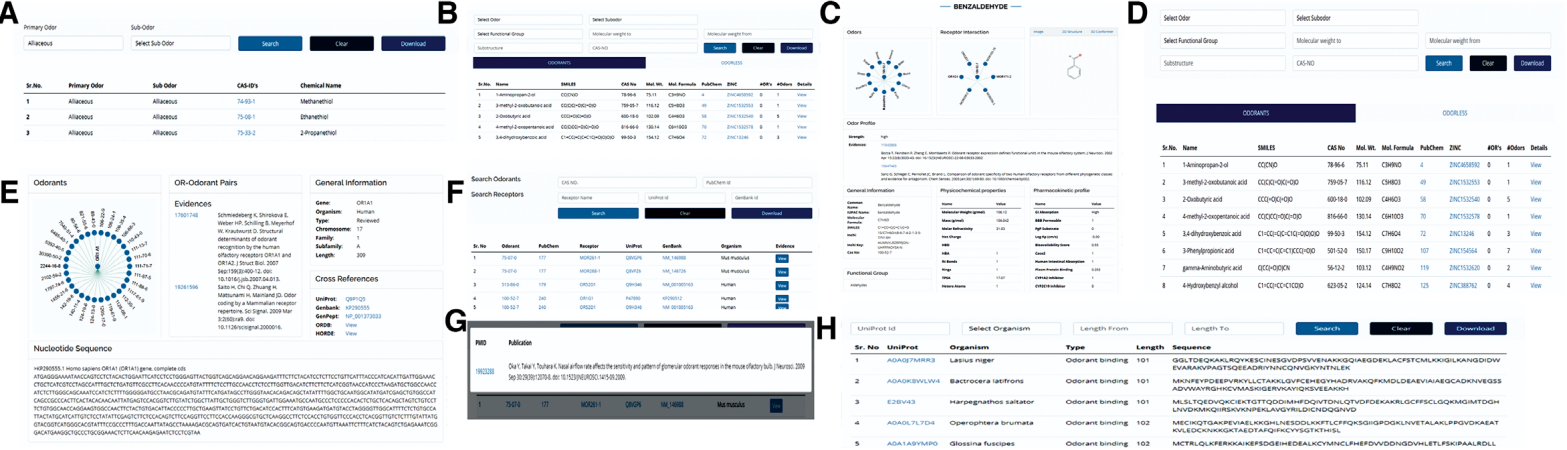
Overview of the OlfactionBase web interface. Snapshot of (**A**) odors page, (**B**) chemical's page, (**C**) detailed information regarding chemical, (**D**) olfactory receptors page, (**E**) detailed information of OR, (**F**) OR−odorant pairs page, (**G**) evidence for each OR−odorant pair, (**H**) information related to OBP, PBP and chemosensory proteins.

**Figure 5.**
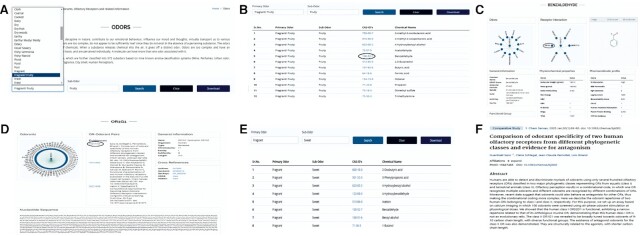
Explore OlfactionBase to look for a ‘fruity’ odor. (**A**) Using the dropdown menu on the odors page, enter the primary odor and sub-odor. (**B**) Search results listing odorants with a ‘fruity’ sub-odor. (**C**) Click on CAS no. gives detailed information about the odorant ‘Benzaldehyde’, including its odors graph, Interacting ORs graph, odor profile, physicochemical parameters, etc. (**D**) Click on OR1G1 node in Interaction graph gives detailed information about the OR1G1 receptor. (**E**) Users can explore different odors linked to ‘Benzaldehdye’ and associated odorants by clicking on any node (‘Sweet’) in the odors graph. (**F**) Experimental evidence of OR−odorant interaction (OR1G1-benzaldehyde).

OlfactionBase provides a user-friendly web interface to browse odorants, odors, olfactory receptors, and odorant-OR pairs. On the ‘Home’ page, users can find a summary of OlfactionBase, including the total number of OR-odorant pairs with evidence for human and *M. musculus*, ORs, chemicals and other proteins. ‘Odors’ page lists out all primary and subodors along with their CAS No. of odorants in a tabular format (Figure 4A). Users can select primary odor and subodor information from the dropdown menus to obtain a list of related compounds. ‘Chemical page list out odorants and odorless compounds in separate tables. General information related to chemicals like Common name, SMILES, CAS No., molecular weight, molecular formula, PubChem ID, ZINC ID, number of interacting ORs and number of odors is given in a tabular format (Figure 4B). By clicking on ‘View’, users can view detailed information about chemicals (odorant/odorless) (Figure 4C). The various search options allow the user to search for a chemical compound within the database based on: (a) CAS No, (b) odors, (c) substructure (SMILES based textual search), (d) functional groups, (e) molecular weight (using lower and upper limit), (f) JSME applet to draw a chemical structure. ‘Receptors’ page contains general information about human and *M. musculus* olfactory receptors like name, organism, length, family, UniProt ID, GenBank ID, number of odorants, etc., in a tabular format (Figure 4D). Users can view nucleotide sequence, protein sequence, and other information related to ORs by clicking on ‘View’ on the ‘Receptors’ page (Figure 4E). The receptor can be searched using various search options given page like: (a) organism, (b) GenBank accession no., (c) chromosome, (d) family and (e) length of the sequence. On ‘OR−odorant pairs’ page, users can find information ORs interacting with odorants (Figure 4F). Related evidences for each OR-odorant pair can be viewed by clicking on the ‘View’ button (Figure 4G). Users can explore proteins other than ORs related to olfaction on the ‘OBP/PBP’ page. It summarises the general information about OBPs, PBPs and chemosensory proteins from 190 species (Figure 4H). User can search for proteins using UniProt ID, organism, and length. All pages are interlinked, enabling users to easily navigate the pages to gain more information about the desired entity. Figure 5 shows how to look for the ‘fruity’ sub-odor and its related odorants, as well as their interacting ORs.

## OLFACTION WHEEL

Various industry-dependent odor classification systems are developed for conveniently learning and remembering the odors in the form of circular diagrams/wheels. These visual odor wheels are constructed to make it easy to understand the relationship between complex and simple odors. The available aroma wheels and work done by Dr Kate Mclean (91) inspired the development of the Olfaction Wheel. Olfaction Wheel, a dynamic circular diagram, shows the inferred relationships among known aroma classification systems, odorants, and how smells are categorized. Odor classification from known classification systems is combined to design an Olfaction wheel, revealing the connection between all classification systems and chemicals possessing the odors. The nine seminal classification systems were chosen from research in established fields: City Smell (92), Compost (93), Drinking Water (94), Fragrance (95), Human Perception (96), Perfumery (97), Urban (98), Waste-water (99) and Wine (100). Odors verbatim not covered in the nine classification systems were added as ‘Other.’ The 106 primary odors were aggregated in alphabetic order, connected to 572 sub-odors, 30 functional groups and 3985 odorants.

**Figure 6.**
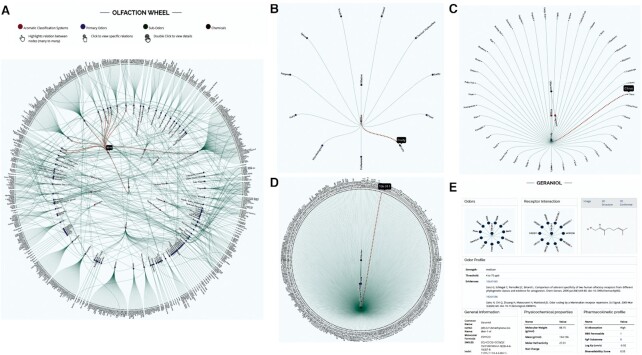
Olfaction Wheel is a dynamic interlinked graph to visualize the relation between primary odors, subodors, and odorants. A single click on the node is for backward or forward navigation, while a double click lands on a page with information about the node in question. (**A**) Olfaction Wheel showing the relation between classification systems (red), primary odors (blue), subodors (green). Mouse hover on Wine highlights the associated primary odors, (**B**) Click on Wine (classification system, red) node generates the subgraph showing its relation to primary odors (blue). (**C**) Click on Fruity (primary odor, green) generates a subgraph showing its linkage to the classification system (red) and sub-odors (blue). (**D**) Click on Citrus (subodor, green) generates a subgraph containing CAS No. (black) of all compounds associated with it. (**E**) Double click on CAS No. (odorant, black) opens up a page containing detailed information about odorants chemical, physiochemical, odor profile, etc.

The olfaction wheel comprises four concentric circles (Figure 6), the innermost circle is derived from compiling information from 10 aroma classification systems. The second and third circles list the primary and subodors and the relation between them. The fourth circle comprises odorants and is linked to sub-odors associated with them. The first three circles (classification system, primary odors, subodors) appear on the Olfaction Wheel homepage, while the fourth circle is visible in the subgraph, which appears on navigating deeper into the graph. All nodes are interlinked, making the graph's exploration (forward and backward) easy to understand visually and conceptually. A single click on a node explores the relationship/ connectivity between the node in question, its parents, and child nodes and helps navigate the wheel (forward and backward). Double click on any node lands on the page containing detailed information related to the node.

## COMPARISON WITH ODORACTOR

OdoRactor (56) and OlfactionBase appear to have overlapping information. OdoRactor is a web server that predicts the ORs for small-molecule compounds by combining two separate techniques; characterising an odorant and then predicting its candidate ORs. However, OlfactionBase is an open-access database that brings together experimental knowledge on different aspects related to olfaction mechanism at one place from a variety of sources, as mentioned above in data compilation section. The data statistics in both databases are shown in Table 2.

**Table 2.** Summary and comparison of OlfactionBase with OdoRactor (56).

Data	Odoractor	OlfactionBase
OR	1608	2067
Odorants	3038	3985
Odorless compounds	0	1124
OR-odorant pair	627	874
Odors	No	Yes (primary odors: 106 sub-odors: 572)
Dynamic representation of odors-odorants relation	No	Yes
Download option	No	Yes

## CONCLUSIONS

The current version of OlfactionBase contains comprehensive information for (a) 3985 odorant compounds, (b) 1124 odorless compounds, (c) 2871 OBP/PBP proteins. It also includes 408 and 466 Odorant-OR interaction pairs for humans and *M. musculus*, respectively. The odorant−OR pairs in OlfactionBase include 197 odorants, 67 receptors, and 419 associations among Humans and 133 ligands, 81 receptors and 518 associations among *M. musculus* (Figure 2). All the data were extracted and annotated from published articles; the majority of data was indeed extracted from the seminal work of Saito *et al.* (69, 83). A typical entry of a chemical compound offers a chemical characterization of the compound, including H-bond donors and acceptors, molecular weight and mass, log *P* and log *K* values among 34 physicochemical properties, 31 pharmacokinetic based properties and five drug-likeness violations. A typical entry for a receptor includes UniProt, GenPept, GenBank accession code, name, FASTA sequence (nucleotide and protein), location, family, and organism; an entry for odors includes primary odor classification and a list of compounds possessing a particular subodor. Moreover, direct links to the corresponding databases (UniProt, PubChem, ZINC, HORDE, ORDB, PubMed) entries are given in each of the entry pages. The database's relational scheme, as shown in Figure 3, on which the current database is based, is specifically designed to capture OR−odorant interaction pairs and odorant−odor preferences as stated in scientific literature and databases.

OlfactionBase offers several navigation and data retrieval options. For compounds, one can perform (a) CAS ID, (b) molecular weight, c) substructure based, (d) odor and (e) functional group-based searches. Similarly, for ORs, (a) GenBank accession number, (b) organism, (c) chromosome, (d) family and (e) length-based search options are available. One can explore compounds possessing specific odors based on primary and sub-group odors. OlfactionBase is a useful and informative resource for investigating odors-odorants and odorant−OR interactions in one place, and it may be a helpful tool for deciphering olfaction mechanisms. It could be valuable for both academic olfaction research and odorant discovery in the industry.

## DATA AVAILABILITY

The website of OlfactionBase is available at https://bioserver.iiita.ac.in/olfactionbase/.
